# Pleural Fluid-to-Blood BNP Ratio May Contribute to Prognosis in Malignant Pleural Mesothelioma

**DOI:** 10.3390/clinpract13050099

**Published:** 2023-09-13

**Authors:** Vasiliki Tsolaki, George E. Zakynthinos, Sotirios Zarogiannis, Paris Zygoulis, Ioannis Kalomenidis, Rajesh Jagirdar, Ioannis Triantafyllou, Konstantinos I. Gourgoulianis, Demosthenes Makris, Epaminondas Zakynthinos

**Affiliations:** 1 Intensive Care Unit, Faculty of Medicine, University of Thessaly, University Hospital of Larissa, 41335 Larissa, Greece; paris.zygoulis@gmail.com (P.Z.); dimomakris@uth.gr (D.M.); ezakynth@yahoo.com (E.Z.); 2 Third Cardiology Clinic, University of Athens, Sotiria Hospital, 11527 Athens, Greece; gzakynthinos2@gmail.com; 3 Department of Physiology, Faculty of Medicine, University of Thessaly, 41500 Larissa, Greece; szarog@med.uth.gr (S.Z.); raj.jagirdar@gmail.com (R.J.); 4 1st Department of Critical Care and Pulmonary Medicine, National and Kapodistrian University of Athens, 10676 Athens, Greece; ikalom@med.uoa.gr; 5 Department of Computer Science and Biomedical Informatics, School of Sciences, University of Thessaly, 35131 Lamia, Greece; itriantafyllou@uth.gr; 6 Respiratory Medicine Department, Faculty of Medicine, University of Thessaly, University Hospital of Larisa, 41335 Larissa, Greece; kgourg@uth.gr

**Keywords:** malignant pleural mesothelioma, survival, brain natriuretic peptide, prognosis

## Abstract

Background: Brain natriuretic peptide (BNP) seems to be produced from malignant mesothelial cells other than cardiomyocytes. We aimed to evaluate whether an increased pleural fluid-to-blood BNP ratio in patients with malignant pleural mesothelioma (MPM) could facilitate prognosis beyond diagnosis. Materials and Methods: Patients with MPM were included (observational study). One- and two-year survival and factors affecting it were tested. To evaluate the prognostic significance of the natriuretic peptide precursor B (NPPB) gene expression in MPM, we constructed a survival curve from data derived from The Cancer Genome Atlas. Results: Nineteen consecutive patients with MPM were included (age: 67 (61, 80), male 78.9%). One- and two-year survival were 52.6% and 31.6%, respectively. Age, performance status, and the other variables tested did not differ between survivors and non-survivors. Non-survivors presented higher pleural fluid BNP in two years (699 (210, 5000) vs. 379.5 (5, 567), *p* = 0.036) and BNP ratios than survivors (1-year: 28.75 (4.05, 150.24) vs. 3.49 (0.3, 26) *p* = 0.001, 2-years: 22.8 (2.42, 150.24) vs. 3.49 (0.3, 7.76), *p* = 0.001). One- and two-year survival rates in patients with BNP ratios above/equal to the median value (8.82) were 20% and 0%, and 88.9% and 66.7%, respectively, in patients with BNP ratios below 8.82 (*p* = 0.006 and *p* = 0.002, respectively). MPM patients with low NPPB expression presented significantly higher survival rates compared to patients with higher expressions (*p* = 0.032). Conclusion: A high pleural fluid/blood BNP ratio, an easily performed in everyday practice, costless biomarker seems to predict poorer survival better than the commonly reported prognostic factors in MPM.

## 1. Introduction

Natriuretic peptides (B-type natriuretic peptide, BNP and N-terminal-pro-BNP, and NT-proBNP) have been routinely used for the diagnosis, prognosis, and therapeutic management of patients with cardiovascular diseases, and mainly, congestive heart failure (CHF) [1]. BNP is considered as being secreted from cardiomyocytes in the left and right heart ventricles as a result of pressure or volume overload [1]. In patients with CHF, increased levels of pleural fluid BNP have been found, correlating to the increased serum levels [2]. In fact, pleural BNP levels are lower than the serum levels, indicating the heart as the origin of the increased serum BNP concentration. This means that pleural concentration results from the molecule’s penetration to the pleura [2]. Thus, although increased pleural fluid natriuretic peptide levels have excellent discriminative properties for the diagnosis of heart failure, their measurement does not add significantly more to the diagnosis than the serum ones [2].

However, BNP has been found to be increased in sepsis, septic shock, and acute respiratory distress syndrome, indicating multiple disorders apart from CHF that may stimulate increased natriuretic peptide secretion in the blood [1,3]. The heart could be implicated as the BNP source production, although this has not been extensively verified. Increased blood BNP values have also been reported in patients with different types of malignancies [4,5,6,7]. Recently, increased BNP levels have been found in the pleural fluid of patients with mesothelioma, in excess of the corresponding serum levels [8]. Pleural fluid/blood BNP ratio with a cutoff value > 2.11 served as a diagnostic marker, with 92% sensitivity and 94.5% specificity for the presence of malignant mesothelioma effusion (*p* < 0.0001), compared to other malignant effusions, parapneumonic effusions, or CHF. This finding indicates that malignant mesothelial cells produce BNP, resulting in an increased pleural fluid-to-blood BNP ratio [8]. This novel finding in the extracardiac production of natriuretic peptides is outstanding considering its diagnostic implications [3,9].

Malignant pleural mesothelioma (MPM) is a rare disease, with a reported incidence of 1–2 per million, although in regions with high asbestos exposure, the cumulative cases are higher [10,11,12]. The prognosis of the disease is poor with an overall reported survival between 9–17 months after diagnosis [13]. Factors that have been reported to affect survival are age, gender, clinical stage, performance status, histological subtype, surgical treatment, and the receipt of chemotherapy [14,15]. C-reactive protein and pleural thickening have also been evaluated [15,16,17,18,19,20]. Recently, malignant mesothelial cell differentiation—and not the histotype—in addition to the chemotherapeutic regimen used and the patient’s response to treatment, were identified as factors affecting survival [21]. Additionally, increased levels of different soluble factors in MPM effusions have been identified to impact survival, beyond the histological subtype [22]. Thus, the MPM secretome, itself, may present valuable information on tumor biology that has not yet been evaluated. Considering that patients present diverse survival rates, despite the MPM histotype, and taking into account our novel finding of BNP production from malignant mesothelial cells, we tried to evaluate whether an increased pleural fluid-to-blood BNP ratio in patients with MPM could facilitate prognosis beyond diagnosis.

## 2. Materials and Methods

This was an observational study conducted in the tertiary University Hospital of Larissa, Greece, between January 2007 and October 2018, including consecutive patients with a diagnosis of malignant pleural mesothelioma. Patients with pleural effusions and evident pleural lesions (mass or pleural thickening) in the computed tomography (CT) scan, who were finally diagnosed with malignant pleural mesothelioma, entered the study. Pleural and serum BNP levels and their corresponding ratio were measured upon the first thoracentesis during the initial assessment of patients with pleural effusion. Only the initial BNP values were considered in the analysis. BNP levels (either pleural or serum) were not re-evaluated after pleuroscopy or induction of chemotherapy. Routine pleural fluid and blood examinations were also performed, while the pleural fluid was sent for cytological analysis. Differential blood cell counting was performed in the Hematology laboratory using automated analyzers, while pleural fluid differential cell count was determined by microscopy using May–Grünwald–Giemsa-stained cytospin slides.

BNP measurements were performed by a commercial analyzer (Triage BNP test; BIOSITE, San Diego, CA, USA).

All the patients underwent a full echocardiographic evaluation of the left and right heart function, according to the Recommendations of the American Society of Echocardiography [23,24].

The performance status was assessed using the Eastern Cooperative Oncology Group Performance Status (ECOG PS) [25]. ECOG performance status ranks performance status on a scale of 0 to 5, according to the patient’s ability to care for themselves, daily activity, and physical ability (walking, working, etc.), according to the following: 0: Fully active, no restrictions on activities. 1: Unable to do strenuous activities, although able to carry out light housework and sedentary activities. 2: Able to walk and manage self-care but unable to work. 3: Confined to bed or a chair for more than 50 percent of waking hours. Capable of limited self-care. 4: Completely disabled. Totally confined to a bed or chair. Unable to do any self-care. 5: death.

Pleural thickness was measured using axial CT images. We measured the maximum tumor thickness perpendicular to the chest wall or mediastinum using axial imaging, according to previous reports [16].

Informed consent was obtained from all the patients, or the next of kin if patients were not alive. The study was conducted in the tertiary University Hospital of Larisa, Greece, between January 2007 and September 2020.

Approval by the Research Ethics Committee of the University Hospital of Larisa was received (2018/35974).

One- and two-year survival was evaluated. Commonly reported variables known to affect survival (age, gender, TNM, pleural thickening, performance status, CRP, albumin, LDH, whole blood cell count, and pleural fluid white blood cells) [14,15,16,17,18,19,20] were tested as prognostic factors. In addition, all three BNP values (pleural fluid, serum BNP, and the pleural fluid/serum BNP ratio) were also tested for prognostication in MPM patients.

### 2.1. Evaluation of Prognostic Significance of NPPB Gene Expression in MPM

To further evaluate the prognostic significance of natriuretic peptide production from the malignant mesothelial cells, we evaluated the diagnostic performance of the natriuretic peptide precursor B (NPPB) gene expression in MPM and constructed a survival curve using data derived from The Cancer Genome Atlas (TCGA) (https://www.cancer.gov/tcga, accessed on 12 March 2019) [26]. For the analysis, we used the PROGgeneV2 Prognostic Database software, which provides a graphic outcome of the survival of patients based on the median value of expression by a given gene [26].

### 2.2. Data Analysis

The normality of the data was tested using the Kolmogorov–Smirnov test. Demographics and fluid characteristics are expressed as median, and the Mann–Whitney U test was used to compare variables. Patients were divided into one- and two-year survivors and non-survivors. Survival was estimated by the Kaplan–Meier analysis and for comparisons between groups, the log-rank test was used.

Moreover, to evaluate the effect of the BNP ratio’s amplitude on survival, the patients were further divided into two subgroups according to the median value of the BNP ratio. One- and two-year survival rates were compared between the two BNP ratio subgroups. Statistical analysis was performed using SPSS 23.0, SPSS, Chicago, IL, USA. Testing was two-sided, with *p* values less than 0.05 considered significant.

## 3. Results

Nineteen consecutive patients (mean age: 67 (61, 80), male 61.5%) with MPM were included in the current study [8]. Demographics and pleural fluid characteristics are presented in Table 1.

The pleural fluid BNP levels were 10 times (even 150 times in certain patients) higher than the corresponding blood levels, at the time of pleural paracentesis (Table 1).

No patient presented any systolic abnormality in the left or right heart on the echocardiography; two patients had Grade I diastolic dysfunction.

One- and two-year survival rates were 52.6% and 31.6%, respectively. Age, TNM stage, performance status, the degree of pleural thickening, LDH, albumin, and CRP did not differ between survivors/non-survivors, thereby showing no prognostic value at all (Table 2). Two-year survivors presented lower WBC (*p* = 0.022). All patients received chemotherapy (without radiotherapy) and none with MPM underwent a surgical resection, according to oncological consultations during the two-year period.

Serum BNP values did not present any significant differences between the two groups. Non-survivors presented higher pleural fluid BNP in two years (699 (210, 5000) vs. 379.5 (5, 567), *p* = 0.036) and BNP ratios than survivors (1-year: 28.75 (4.05, 150.24) vs. 3.49 (0.3, 26) *p* = 0.001, 2-years: 22.8 (2.42, 150.24) vs. 3.49 (0.3, 7.76), *p* = 0.001) (Table 2).

**Figure 1:**
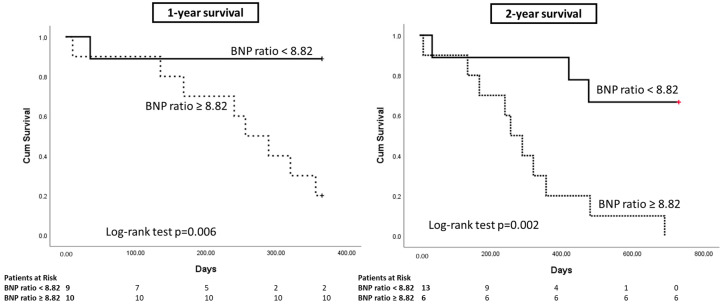
One- and two-year survival rates in patients with malignant pleural mesothelioma. Stratification of the patients according to the 50th percentile BNP ratio (8.82). Overall, 1-year survival was 52.6% and 2-year survival was 31.6%; patients with BNP ≥ 8.82: 1-year survival rate 20%, 2-year survival 0%; patients with BNP < 8.82: 1-year survival rate 88.9%, 2-year survival 66.7%; log-rank: *p* = 0.006 and *p* = 0.002, respectively.

Survival was further estimated with stratification of the patients, according to the BNP ratio. One- and two-year survival rates in patients with BNP ratios equal to or above the median value (8.82) were 20% and 0%, respectively. In contrast, the survival of patients with BNP ratios below 8.82 were: 88.9% and 66.7%, respectively (Figure 1).

Only one patient presented a BNP ratio < 1. This patient (BNP ratio: 0.3) was alive for 6330 days after the initial malignant mesothelioma diagnosis. In fact, this is the only patient on whom an MPM diagnosis had been performed 5 years before the pleural and serum BNPs were measured. Repeat thoracentesis was performed due to recurrent pleural effusion. Follow-up CT scans (10 years after initial diagnosis) revealed contralateral pleural effusions and ascites of malignant mesothelioma origin. The patient underwent surgical resection of the peritoneal masses before finally receiving chemotherapy.

In the majority of the patients, a histopathological diagnosis was not obtained; the diagnosis was based on pleural fluid cytology. Only three patients underwent a pleuroscopy biopsy; all were diagnosed with the epithelioid histotype. These patients presented diverse BNP ratios and survival rates (pleural/blood BNP ratios: 2.42 (survival 476 days), 22.82 (survival 256 days), 150.24 (survival 135 days)).

**Figure 2:**
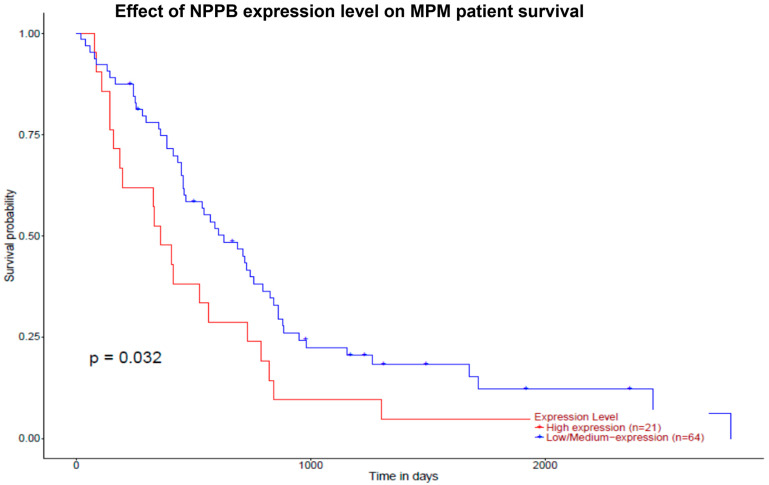
Effect of NPPB expression level on the survival of patients with malignant pleural mesothelioma. Results of Kaplan–Meier survival analysis from The Cancer Genome Atlas (TCGA) mesothelioma dataset derived from the PROGgeneV2 database show that low NPPB gene expression favors survival (shown in days) in MPM patients. NPPB: natriuretic peptide precursor B; MPM: malignant pleural mesothelioma.

Analysis of the NPPB gene expression in MPM showed that the MPM patients with low NPPB expression presented significantly higher survival rates compared to patients with higher expressions (*p* = 0.032; Figure 2).

## 4. Discussion

Pleural BNP levels have been mainly used in the diagnosis and prognosis of patients with heart failure [3]. However, in this disease category, the increased pleural BNP levels reflect the blood ones, as they result from vascular leakage to the pleural cavity. BNP is produced primarily by cardiomyocytes and released in the circulation in response to strain on the heart wall [1,9]. In this respect, the pleural fluid/blood BNP ratio has been consistently found as below the value of 1 [2,3,27]. We have recently found that the pleural fluid/blood BNP ratio may exert the value of one (reaching values up to 150) in patients with MPM [4]. Moreover, it seems that the pleural fluid/blood BNP ratio may facilitate prognosis, and actually, more accurately than commonly used variables in MPM. Therefore, the current study presents additional information, in accordance with our previous report, concerning the novel finding on the production of BNP by malignant mesothelial cells: survival of MPM patients appears worse as the extent of the pleural fluid-to-blood BNP ratio increases.

The disproportionately greater BNP concentration in the pleural fluid, compared to the corresponding blood sample, was an accurate diagnostic tool for the discrimination of patients with MPM from other types of effusions (non-mesothelioma malignant effusions, parapneumonic effusions, and transudates) [8]. Following a Bayesian approach, pleural fluid BNP levels that, at a minimum, are double those in the blood, appear to offer very strong evidence for ruling in favor of mesothelioma [8,9].

Serum BNP has been widely used in the diagnosis of CHF [28]. Pulmonary hypertension and acute cor pulmonale, resulting from a massive pulmonary embolism, may lead to increased BNP levels. Additionally, RV overload, leading to myocardial wall stretch, is the main factor driving the increased BNP production [1]. Increased BNP levels (>1000 ng/mL) have also been reported in patients with septic shock, correlating with the presence of septic cardiomyopathy. Biventricular dysfunction resulting from proinflammatory cytokine action on the myocardium, volume overload as a result of resuscitation protocols, and impaired renal function, alongside ARDS and mechanical ventilation, ultimately, lead to RV dysfunction, have all been considered as factors that lead to BNP overproduction [3] In severe sepsis, increased BNP levels and poorer outcomes have been attributed to cardiac involvement [29]. Therefore, one might question whether CHF was also present in some of our patients, considering the serum BNP levels. In three patients, serum BNP exceeded the cutoff value of 100 pg/mL, which has been extensively used to diagnose heart failure [1], although less than 400 pg/mL (BNP in the range of 100–400 pg/mL is considered the “gray zone” levels for the diagnosis of CHF) [1]. In those, the patient’s pleural/blood BNP ratios were 2.4, 26, and 18.7, respectively, i.e., pleural BNP was much higher than blood levels. Moreover, none of our patients presented more than mild diastolic heart failure. Hence, cardiac involvement in these patients was essentially ruled out. Even in the patient with a serum BNP value greater than the pleural, the serum BNP level was only 16 pg/mL, meaning that CHF was excluded. Increased BNP production has been reported in patients with malignancies (urothelial carcinoma, solid or hematological malignancies) without signs of CHF [4,5]. Yet, in one series, better control of the volume status led to a substantial decrease in natriuretic peptide levels [6]. The presence of cardiac metastasis has not been extensively evaluated in these patients presenting with increased BNP levels; BNP has been found to be a sensitive marker denoting cardiac metastasis in patients with non-small-cell lung cancer (N-SCLC) [7]. On the contrary, in our patients with MPM, serum BNP was not elevated, nor was it increased in the patients with other lung malignancies [8]. The production of BNP from malignant mesothelial cell lines in vitro and the increased BNP levels in the pleural fluid, only from patients with MPM and no other lung malignancies, was a novel finding [8]. Detectable BNP mRNA has been found in small-cell lung cancer cell lines, yet malignant pleural effusion is mainly found in N-SCLC [30,31]. These data suggest that further studies should focus on the malignant cell secretome.

Apart from the diagnosis, the present study indicates that significant localized pleural BNP production in patients with MPM denotes worse prognoses (Figure 1). This is in accordance with the data of The Cancer Genome Atlas data, which shows that MPM patients with higher expression of the NPPB gene present a worse overall survival (Figure 2) [27]. It is reasonable that increased NPPB expression from MPM cells leads to increased local BNP production, resulting in pleural fluid levels that are higher than the serum ones. Similarly, in the setting of CHF, NPPB expression is strongly increased in the ventricular myocardium of the heart, resulting in increased NT-pro-BNP and BNP levels in the blood [32]. Of course, this is an assumption that requires further elucidation. However, strengthening our hypothesis, in our clinical study the patients presented rather low serum BNP levels (median value < 100 ng/mL—indicating no heart involvement), while the corresponding pleural fluid BNP level was at least more than double [8]. Interestingly, the highest pleural fluid BNP value (also indicated by an increased pleural fluid-to-blood BNP ratio probably related to the NPPB overexpression from MPM) denoted the worst survival.

Currently, stage and histology are the strongest prognostic factors among patients with mesothelioma. However, histological confirmation is often elided when a diagnosis is reached using cytological analysis. In the present analysis, only three patients had histologic confirmation, all of them with an epithelioid histotype, which is the most common among mesothelioma [21]. The performance status, age > 75, elevated LDH, and hematologic abnormalities are variables traditionally used for prognostication [23]. Women have recently been identified to present better survival, as also reported in our study. Less asbestos exposure burden, sex hormones, and different tumor biology have been proposed as possible contributors [33]. Increased CRP value has also been proposed as a marker denoting poorer survival, although cumulative reports have focused on the value of pleural lesion thickness to predict survival [17,18,19,20]. In our study, all the above-mentioned variables did not differ between survivors and non-survivors. We assume that the small sample size precluded the identification of significant differences. In patients with MPM and a high concentration of soluble cytokines, survival is worse, irrespective of histology and performance status, meaning that other commonly identified factors affect the outcome in these patients; the pleural fluid/blood BNP ratio could serve as one [21].

Although a higher BNP ratio denotes a poorer prognosis, BNP seems to have a protective role through its antifibrotic, antimitotic, and anti-inflammatory properties [5,34,35,36,37]. Shared mesodermal embryonic origin of mesothelial cells with cardiomyocytes could serve as a possibility for the potential to secrete BNP under special circumstances [38,39]. Dynamic mesothelial cell transition potential may result in different phenotypes; the differentiation into myofibroblasts may contribute to pleural thickening and rind formation (a significant feature in mesothelioma). Accordingly, myofibroblasts, in animal studies, have been found to synthesize BNP, probably in a way, to act as a local anti-fibrotic agent [5,34,35]. Extended fibrosis may lead myofibroblasts to produce greater amounts of BNP, although the former could be related to a poorer prognosis. Similarly, it has been shown that BNP inhibits the production of IL-1β, one of the central mediators in inflammation and immunity, thereby contributing to the restoration of cytokine production [36]. Additionally, anti-mitotic actions have been proposed for natriuretic peptides [36,37]. Therefore, increased BNP production from malignant mesothelial cells may reflect an attempt to limit the inflammation and mitosis imposed by the malignant burden. In accordance with that hypothesis, in vitro BNP production was the highest in the least aggressive MPM histotype (epithelioid) and lowest in the biphasic type [8]. We suppose that, clinically, as the malignant load increases (worsening the prognosis), pleural BNP increases as well, to act protectively, by trying to limit the damage. Unfortunately, the MPM histotype was only known in three patients; in all, the epithelioid histotype (the most frequent) was diagnosed. However, in these three patients, the BNP ratios were diverse and a poorer prognosis was present as the ratio increased. However, whether BNP production modulates pleural fibrosis or influences tumor cell growth is purely speculative. These are only preliminary results and further studies are needed to combine the histopathologic diagnosis and malignant load to the BNP ratio. This may decode the purpose of malignant mesothelioma cell BNP production.

The present study included a small study of MPM patients, although above the cutoff value of 10 patients that is needed to avoid bias when estimating sensitivity and specificity [40]. In addition, the analysis was based on a dimensionless number (BNP ratio), which is not affected by a reference standard. Regarding the blood BNP values, although there were three patients in the “grey zone” for CHF when considering blood BNP values [1], the corresponding pleural fluid BNP concentrations were higher than the serum ones. Thus, we provokingly assume the opposite from the well-identified BNP kinetics, whereby the peptide enters the blood from the pleural cavity. Moreover, a diagnosis of CHF was rigorously excluded by a full echocardiographic evaluation. The inclusion of MPM patients with CHF would have been very interesting. In this scenario, where there is increased production from both the pleural cavity and heart, the BNP kinetics and interaction between the levels in the pleural cavity and serum might be rather unpredictable and would have been intriguing to evaluate. Another limitation is that the histological subtype has not been evaluated apart from the three patients, all of whom presented the epithelioid histotype. Recently, Guzman-Casta et al. reported that 95.6% of the 136 MPM patients included in their study were diagnosed with the epithelioid histotype. Diverse survival rates were reported among them, depending on the cell differentiation and the response to treatment, while in a second study, the MPM secretome drove survival, irrespective of histology [21,22]. These results highlight that other factors, besides the mesothelioma histotype, may influence survival. The BNP ratio could add valuable information concerning the prognosis of MPM patients. We suppose that it would be very interesting in the future to combine BNP ratios with the histological subtype in terms of prognosis. Certainly, our preliminary results in this small study group should be corroborated in future studies, which include higher numbers of patients, in an effort to increase the robustness of our findings.

## 5. Conclusions

In conclusion, in the present study on malignant mesothelioma patients, the evaluation of the pleural fluid-to-blood BNP ratio, at the initial assessment, was found to discriminate between survivors and non-survivors; the higher the value, the worse the survival was. The BNP ratio indicates a significant prognostic element, easily performed in everyday clinical practice, from the already available pleural fluid drawn for diagnosis, and with a rather low cost. The focus of future research should encompass the MPM secretome, as it may decode hidden information that may change our understanding of tumor biology and kinetics.

## Figures and Tables

**Table 1:** Demographic characteristics and fluid data.

	Malignant Mesothelioma Effusions (MME) (*n* = 19)
Age	67 (61, 80)
Sex (male)	15 (78.9%)
Smoking habits/current smokers	17 (89.5%)/16/17 (94%)
Smoking habits (pys)	45 (0, 100)
BMI (kg/m2)	25.5 (22.4, 34)
Arterial hypertension	8 (42%)
Diabetes mellitus	4 (21%)
Asbestos exposure	
Definite/confirmed	10 (52.6%)
Possible	5 (26.3%)
Unknown	4 (21%)
TNM	3 (3, 4)
Pleural thickness (max) (cm)	3 (1.2, 4.5)
Performance status	2 (1, 3)
White blood cells (blood) (/μL)	8900 (5600, 12,900)
CRP (mg/dL)	3.2 (1.2, 4.3)
Albumin (serum) (mg/dL)	3.8 (3.2, 4.3)
Serum creatinine (mg/dL)	1.19 (1.04, 1.4)
White blood cells (pleural fluid) (/μL)	2890 (100, 13,000)
Lymphocytes	70% (56, 80%)
Neutrophils	5% (0, 24%)
Mesothelial cells	20% (10, 35%)
Pleural fluid BNP (pg/mL)	457 (5, 5000)
Whole blood BNP (pg/mL)	73 (8, 268)
BNP ratio	8.82 (0.3, 150.24)
Pleural fluid LDH (IU/mL)	429 (100, 1150)
Serum LDH (IU/mL)	231 (127, 2245)
LDH ratio	1.7 (0.2, 4.7)
Pleural fluid protein (mg/dL)	4.3 (2.9, 5.2)
Blood protein (mg/dL)	6.9 (6.1, 7.8)
Protein ratio	0.63 (0.48, 0.79)

Data are presented as median (minimum, maximum). BMI: body mass index; BNP: brain natriuretic peptide; LDH: lactate dehydrogenase; pys: packs per year; TNM: tumor node metastasis staging system. The ratio (for BNP, LDH, and protein) was calculated as the ratio of the level in the pleural fluid to the level in blood/serum.

**Table 2:** Comparisons between survivors and non-survivors (in one and two years).

	1-Year Survival		2-Year Survival	
	Survivors (*n* = 10)	Non-Survivors (*n* = 9)	Survivors (*n* = 6)	Non-Survivors (*n* = 13)
Age	66.5 (61, 78)	68 (62, 80)	65.5 (61, 70)	68 (62, 80)
Sex (male)	7 (70%)	8 (88.9%)	5 (83.3%)	10 (76.9%)
Smoking habits/current smokers	9/10 (90%) 9/9 (100%)	8/9 (88.9%) 7/8 (87.5%)	5/6 (83.3%) 5/5 (100%)	12/13 (92.3%) 12/12 (100%)
Smoking habits (pys)	45 (0, 50)	30 (0, 100)	46.5 (0, 50)	40 (0, 100)
BMI (k/m^2^)	25.9 (22.4, 34)	25.3 (23, 27.4)	25.6 (22.4, 34)	25.5 (23, 32)
Arterial hypertension	5 (50%)	3 (33.33%)	2 (33.4%)	6 (46.2%)
Diabetes mellitus	3 (30%)	1 (11%)	2 (33.4%)	2 (15.4%)
Asbestos exposure				
Definite/confirmed	4 (40%)	6 (66.67%)	3 (50%)	7 (53.8%)
Possible	3 (30%)	2 (22.2%)	1 (16.7%)	4 (30.8%)
Unknown	3 (30%)	1 (11.1%)	2 (33.3%)	2 (15.4%)
TNM	3 (3, 4)	3 (3, 3)	3 (3, 4)	3 (3, 4)
Pleural thickness (max) (cm)	2.8 (1.5, 4.3)	3.4 (1.2, 4.5)	3.1 (1.8, 4.3)	3 (1.2, 4.5)
Performance status	1 (1, 2)	2 (1, 3)	1 (1, 2)	2 (1, 3)
White blood cells (blood) (/μL)	8680 (5600, 10,200)	9870 (6700, 12,900)	7339.5 (5600, 95,670) *	9379 (6700, 12,900)
CRP (mg/dL)	3.2 (1.2, 4.3)	3.3 (2.8, 3.8)	2.6 (1.2, 4)	3.4 (2.8, 4.3)
Albumin (serum) (mg/dL)	3.7 (3.2, 4.3)	3.8 (2.8, 3.8)	3.8 (3.2, 4.3)	3.7 (3.4, 4.2)
White blood cells (pleural fluid) (/μL)	2329 (100, 9400)	2980 (800, 13,000)	2329 (100, 9400)	2980 (200, 13,000)
Lymphocytes	78% (60, 80%) *	65% (56, 72%)	70% (60, 80%)	70% (56, 80%)
Neutrophils	7.5% (2, 24%)	5% (0, 10%)	15% (4, 24%) *	4% (0, 10%)
Mesothelial cells	16.5% (10, 20%) #	30% (20, 35%)	15% (15, 20%) *	28% (10, 35%)
Pleural fluid BNP (pg/mL)	413 (5, 5000)	1016 (210, 5000)	379.5 (5, 567) *	699 (210, 5000)
Serum BNP (pg/mL)	108.5 (16, 203)	26.5 (8, 268)	86 (16, 110)	58 (8, 268)
BNP ratio	3.49 (0.3, 26) *	28.75 (4.05, 150.24)	3.49 (0.3, 7.76) *	22.8 (2.42, 150.24)
Pleural fluid LDH (IU/mL)	404 (100, 1134)	459 (182, 1150)	418 (100, 489)	445 (114, 1150)
Serum LDH (IU/mL)	219 (127, 2245)	245 (151, 380)	173 (155, 207)	245 (127, 2245)
LDH ratio	1.6 (0.2, 3.9)	1.87 (0.8, 4.7)	2.4 (0.5, 3.2)	1.35 (0.2, 4.6)

*: *p* value < 0.05, #: *p* value < 0.001, comparisons were made between 1-year survivors and non-survivors and 2-year survivors and non-survivors. BMI: body mass index; BNP: brain natriuretic peptide; CRP: C-reactive protein; LDH: lactate dehydrogenase; pys: packs per year; TNM: tumor node metastasis classification system.

## Data Availability

Data concerning the patients can be available upon reasonable request. For the analysis of the Natriuretic Peptide Precursor B (NPPB) gene expression in MPM data derived from The Cancer Genome Atlas (TCGA) NPPB gene expression were used.
